# Supplementary material for: Subcellular mRNA kinetic modeling reveals nuclear retention as rate-limiting
Source: Mol Syst Biol. 2024 Nov 15;20(12):1346–71. doi: 10.1038/s44320-024-00073-2 (PMC11611909; doi:10.1038/s44320-024-00073-2)
Supplement: Supplementary file 1 — Appendix [file 44320_2024_73_MOESM1_ESM.pdf]

Appendix for

Subcellular mRNA kinetic modeling reveals nuclear retention as rate-limiting

Table of contents

Appendix Figure S1 2
Appendix Figure S2 3

Appendix Figure S1

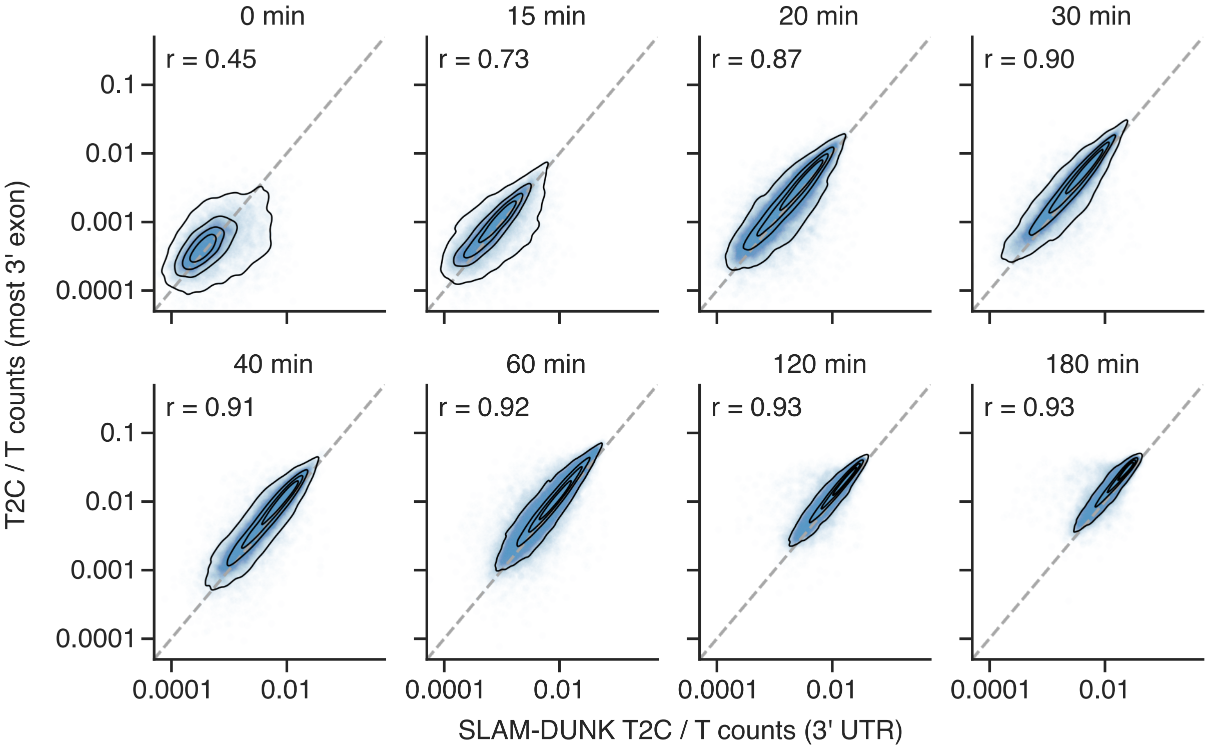

Appendix Figure S1. Mapping and T2C counting comparison to SLAM-DUNK method

Scatterplots of T2C/T count data of all expressed transcripts from nuclear, cytosolic and membrane samples, faceted by timepoints. Labeling data is obtained using our custom analysis pipeline (y-axis, only data from most 3' exon per transcript) and the established SLAM-DUNK method (x-axis), where alignment is done on the 3' UTR only. Spearman rank correlation is shown on top left in each facet. Number of data points from top left (t = 0 min) to bottom right (t = 180 min): n = 10897, n = 13040, n = 42836, n = 26550, n = 28657, n = 69195, n = 36652 and n = 36601. Most 3' exon T2C and SLAM-DUNK 3' UTR data was filtered to contain at least a T coverage of 1000 and 3000, respectively. Overall, there is very high agreement between the methods and no systematic over- or underestimation visible. For the t=0 min samples the agreement is lower, since the T2C mutations are largely sequencing errors.

Appendix Figure S2

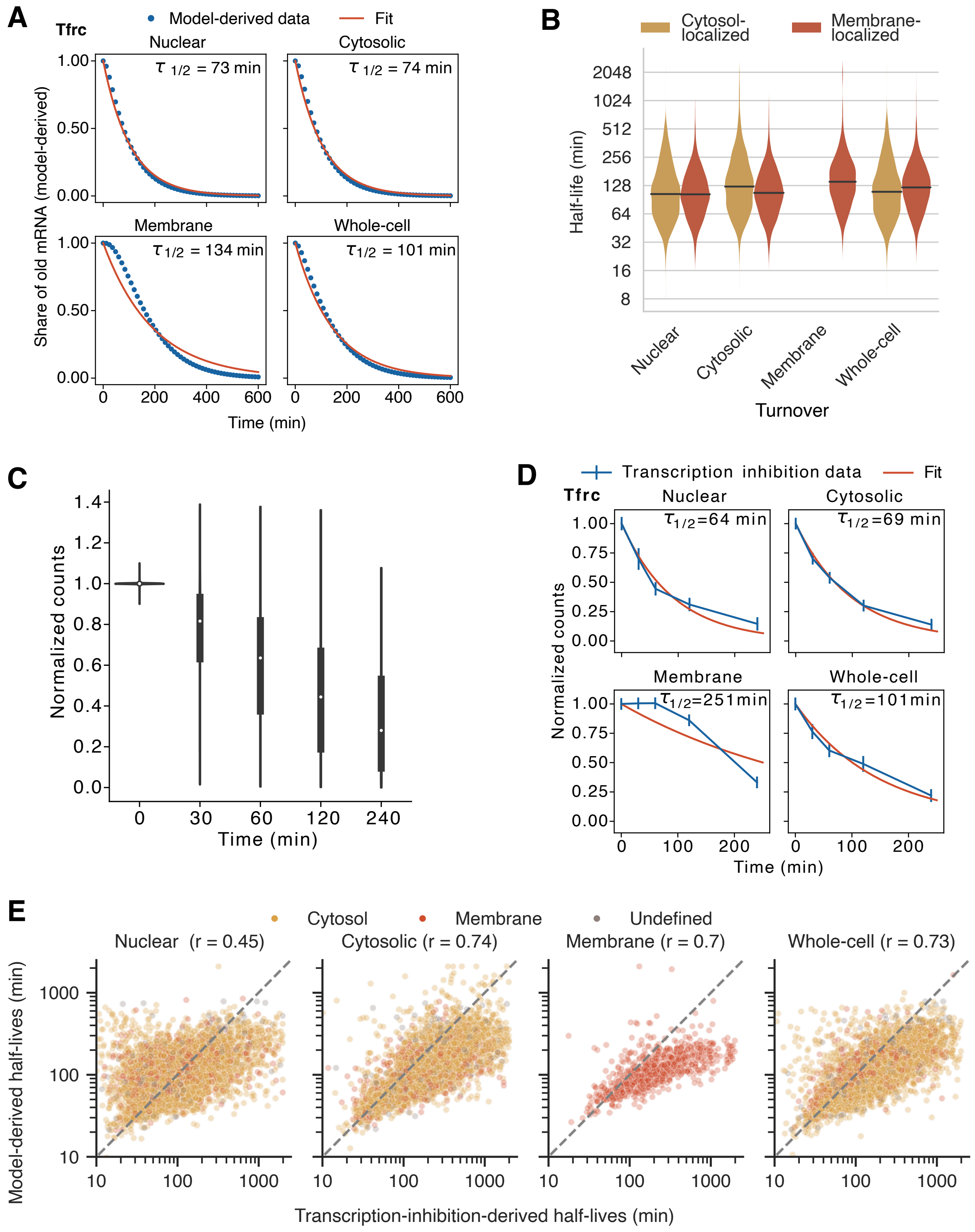

Appendix Figure S2. Estimating model-derived pulse-chase-like and transcription inhibition-derived mRNA half-lives

A. Exemplary fit to determine model-derived, aggregated half-lives, shown for Tfrc. From the subcellular rates, model-derived pulse-chase trajectories are calculated for different compartments (blue dots). To this data, a simple exponential decay model is fit (red line). Best fit values are shown on top right in each parameter facet.

B. Violin plot showing aggregated half-lives of model-derived parameters for cytosol- (yellow, n = 7677) and membrane-localized (red, n = 1693) transcripts.

C. Combined violin and box plots of normalized counts from transcription inhibition experiment using Flavopiriodol (n = 37297, all compartments). x-axis shows time exposed to flavopiridol. White dots depict median values. Lower and upper hinges of box plots correspond to the 25th and 75th percentiles, respectively.

D. Exemplary fit to determine transcription inhibition-derived half-lives, shown for Tfrc. A simple exponential decay model (red line) was fitted to normalized counts (n = 3 replicates per time point, blue vertical lines indicate standard error). Best fit values are shown on top right in each parameter facet.

E. Scatterplots of model-derived and transcription inhibition-derived aggregated half-lives of nuclear (n = 5979), cytosolic (n = 5910), membrane (n = 811) and whole-cell (n = 6316) compartments. Color indicates localization. Spearman rank correlation is shown in facet title. Dashed, gray line is the identity line.
