# Supplementary material for: Subcellular mRNA kinetic modeling reveals nuclear retention as rate-limiting
Source: Mol Syst Biol. 2024 Nov 15;20(12):1346–71. doi: 10.1038/s44320-024-00073-2 (PMC11611909; doi:10.1038/s44320-024-00073-2)
Supplement: Supplementary file 14 — Expanded View Figures [file 44320_2024_73_MOESM14_ESM.pdf]

Expanded View Figures

Figure EV1. Overview of mRNA expression and T2C labeling data.

(**A**) Differential expression analysis of whole-cell extract RNA sequencing data. Volcano plots showing BH-adjusted *p* values against fold changes logarithmized to base 2. Facets show subsequent 4sU labeling times tested against either t=0 min labeling for the high 4sU dosage (500 µM) or t = 60 min labeling for the low 4sU dosage (100 µM). Differentially expressed genes (padj < 0.05 & log2fc > 0.5, indicated by horizontal and vertical dashed lines) are shown as red dots and stable genes are shown as black dots. Brackets indicate that both subcellular and whole-cell samples of the time point and 4sU concentration tested in the facet were excluded from further analysis. Considering only data included in our main analysis, only one gene, *Kantr*, is differentially expressed and was removed from subsequent analyses. (**B**) Gene expression in subcellular fractions. Histograms show TPM values averaged over all time points and replicates for nuclear, cytosolic and membrane fractions. Vertical dashed lines indicate an average TPM of 1.5. If a gene has an average TPM >1.5 in at least one subcellular fraction, it is considered expressed. (**C**) Mitochondrial contamination of the nuclear fraction. The ratio of the summed counts from all mt-DNA-encoded over all nuclear-encoded transcripts is shown for all samples (black dots) split by fraction. Orange lines depict the median across samples. (**D**) Box plot of ratio between membrane and cytosolic expression (membrane enrichment) with transcripts classified by encoded TS as in Fig. 4C. Classification from left to right: cytosol-localized transcripts with no TS (n = 6262), nuclear DNA-encoded mitochondrial proteins (n = 763 with 661 being cytosol-localized), transcripts with tail-anchored transmembrane proteins (n = 78), membrane-localized transcripts with no known TS (n = 531), transcripts encoding signal peptides (n = 310) or transmembrane helices (n = 956) or both (n = 47). Center lines of box plots depict the median values. Notches show the 95% confidence interval of median values acquired through boot-strapping (n = 1000). Lower and upper hinges of box plots correspond to the 25th and 75th percentiles, respectively. (**E**) Estimates of T2C conversion rate. Conversion rates of individual samples are plotted over 4sU labeling time. Conversion rates were estimated fitting a Binomial mixture model to T and T2C count data of each sample. Color indicates compartment. Shape indicates 4sU concentration. Conversion rate increases for high dosage (roughly two-fold increase from 15 to 60 min), but stays constant for low dosage. (**F**) Comparison between samples labeled with 500 µM or 100µM 4sU for 60 min. Boxes show the share of new to total mRNA in subcellular compartments with colors indicating specific replicates. Center lines of box plots depict the median values. Lower and upper hinges of box plots correspond to the 25th and 75th percentiles, respectively. Although 4sU conversion rates are different between samples, see (**D**), the share of new mRNA is similar. (**G**) Principal component analysis of the T2C labeling data using 2,000 most variable genes. Samples cluster by exposure time to 4sU rather than replicate, with nuclear pre-mRNA being separated from other compartments. Source data are available online for this figure.

 © The Author(s)

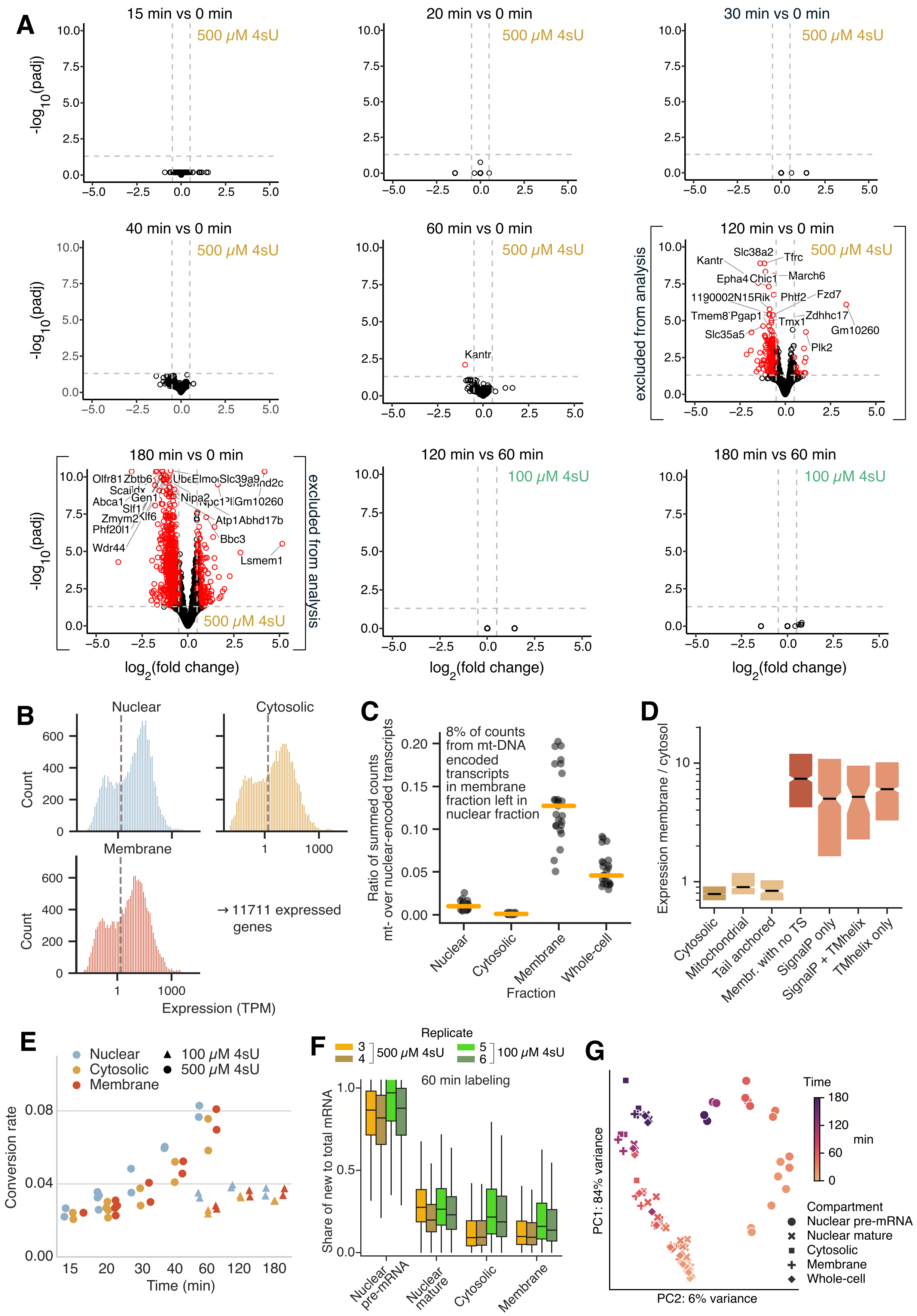

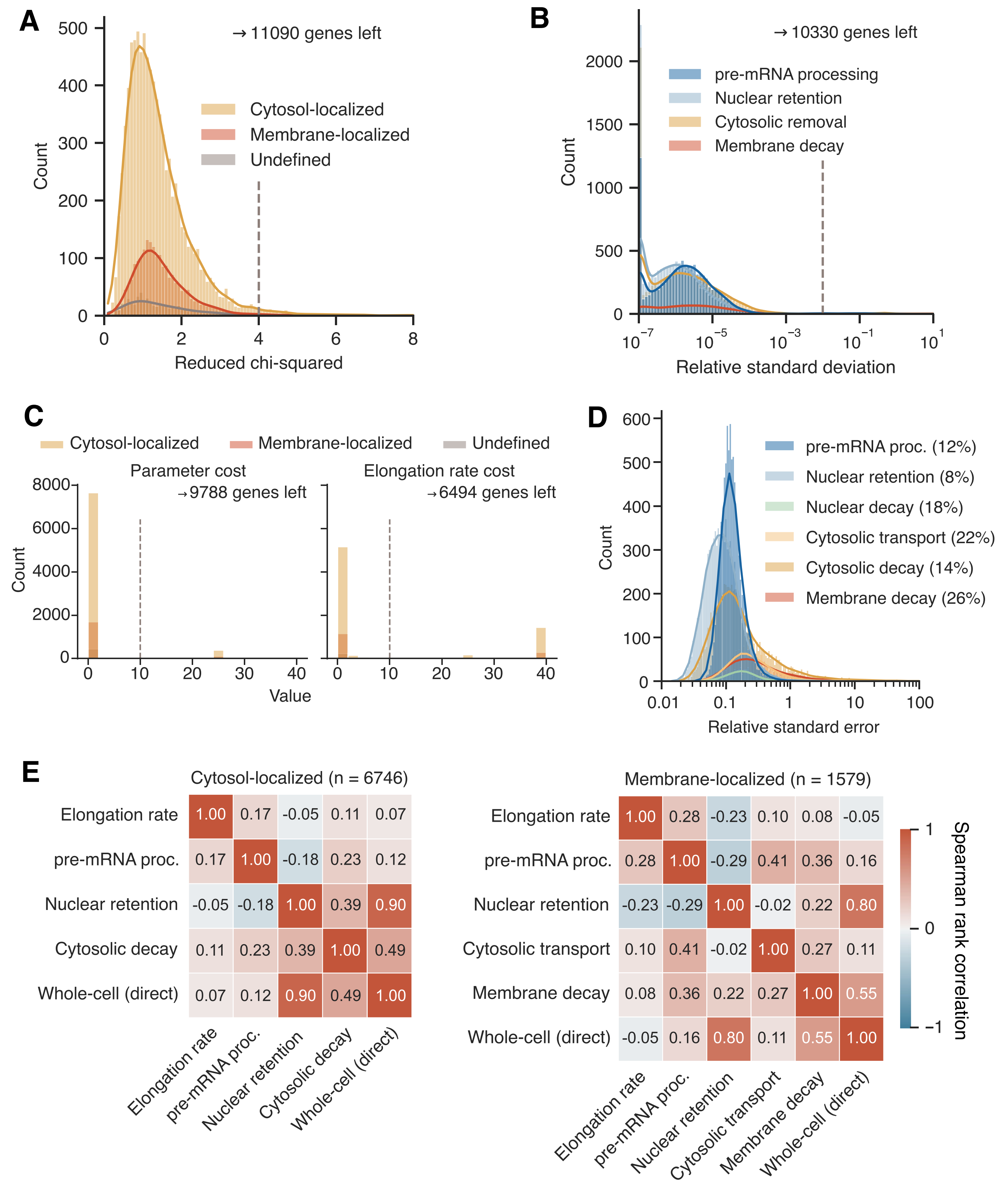
A
→ 11090 genes left
Cytosol-localized
Membrane-localized
Undefined
Count
Reduced chi-squared
B
→ 10330 genes left
pre-mRNA processing
Nuclear retention
Cytosolic removal
Membrane decay
Count
Relative standard deviation
C
Cytosol-localized
Membrane-localized
Undefined
Parameter cost
→9788 genes left
Elongation rate cost
→6494 genes left
Count
Value
D
pre-mRNA proc. (12%)
Nuclear retention (8%)
Nuclear decay (18%)
Cytosolic transport (22%)
Cytosolic decay (14%)
Membrane decay (26%)
Count
Relative standard error
E
Cytosol-localized (n = 6746)
Elongation rate 1.00 0.17 -0.05 0.11 0.07
pre-mRNA proc. 0.17 1.00 -0.18 0.23 0.12
Nuclear retention -0.05 -0.18 1.00 0.39 0.90
Cytosolic decay 0.11 0.23 0.39 1.00 0.49
Whole-cell (direct) 0.07 0.12 0.90 0.49 1.00
Membrane-localized (n = 1579)
Elongation rate 1.00 0.28 -0.23 0.10 0.08 -0.05
pre-mRNA proc. 0.28 1.00 -0.29 0.41 0.36 0.16
Nuclear retention -0.23 -0.29 1.00 -0.02 0.22 0.80
Cytosolic transport 0.10 0.41 -0.02 1.00 0.27 0.11
Membrane decay 0.08 0.36 0.22 0.27 1.00 0.55
Whole-cell (direct) -0.05 0.16 0.80 0.11 0.55 1.00
Spearman rank correlation

 © The Author(s)

Figure EV2. Quality control of fit results.

(**A**) First quality control step. Histograms of reduced chi-squared, which is minimized during fitting (optimal value is 1). Vertical dashed line shows cutoff of 4. If the best fit for a transcript has a value higher than the cutoff, the transcript is excluded. (**B**) Second quality control step. Histograms of relative standard deviation, calculated by dividing the standard deviation of the ten best-fit results by the value of the best fit. All values < 1e-07 were set to 1e-07. Vertical dashed line shows cutoff of 0.05. If the relative standard deviation for a transcript has a value higher than the cutoff, the gene is excluded. (**C**) Third quality control step. Histograms of the boundary cost for all kinetic parameters (left) and elongation rate (right). Boundary cost is near 0 if fit value is far away from upper and lower limits and increases drastically as the value approaches the allowed limits. A high boundary cost therefore indicates that the fit was stuck at maximum or minimum allowed value. Vertical dashed line shows cutoff of 10. If the best fit for a transcript has a parameter cost higher than the cutoff, the transcript is excluded. Transcripts with a elongation rate cost higher than the cutoff are only excluded for analyses specifically regarding the elongation rate. Colors indicate mRNA localization. (**D**) Accuracy of parameter estimations. Histograms of the relative standard error colored by parameter. Relative standard errors are the square root of the diagonal elements of the inverse Hessian matrix (1σ uncertainty output by lmfit) divided by the corresponding parameter value. Median relative standard errors of each parameter are shown in brackets as percentages in the figure legend. 75% of parameter estimates have a relative error smaller than 19%. (**E**) Heatmaps showing the Spearman rank correlation between kinetic parameters for cytosol- (left) and membrane-localized (right) transcripts. Source data are available online for this figure.

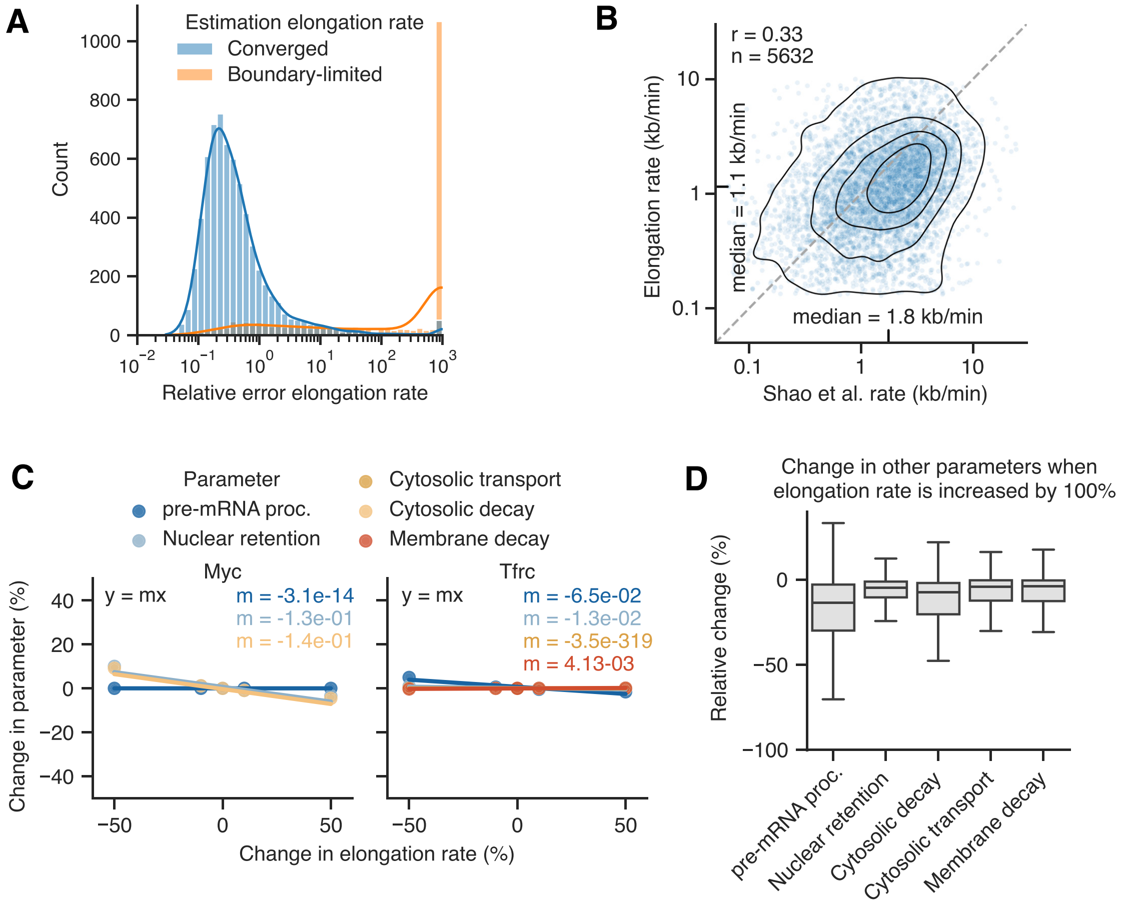

Figure EV3. Quality control of the transcript elongation rate.

(**A**) Accuracy of elongation rate estimations. Histogram of the relative standard error of the elongation rate. Relative standard errors are the square root of the diagonal elements of the inverse Hessian matrix (1σ uncertainty output by lmfit) divided by the corresponding parameter value. 75% of the converged fits (n = 6496) have a relative error smaller than 63% with an overall median of 30%. For boundary-limited estimates (n = 2007) errors were high or could not be determined. For illustration purposes, here, relative standard errors containing infinities or NAs were set to 1000. (**B**) Comparison of estimated transcription elongation rate to published data (Shao et al, 2022). Scatterplot shows converged elongation rates and from Shao et al measured in serum-naive state mESCs. Spearman rank correlation is shown on top left. Black lines are 2-dimensional KDE to indicate density of points. Dashed, gray line is the identity line. (**C**) Sensitivity analysis for the elongation rate parameter. Regression plots for transcripts of two exemplary genes, *Myc* and *Tfrc*, are shown. For all multiple-exons transcripts that do not show nuclear decay, we repeated the fitting procedure fixing the elongation rate at -50%, -10%, +10% and +50% of its best-fit value and initializing with the best-fit values of the remaining parameters. Then, for each transcript and parameter, we fitted a linear regression $y = mx$, where y is the change in the fitted parameter and x is the fixed relative change of the elongation rate. The slope m gives a measure of how much a parameter is influenced by changes in the elongation rate. (**D**) Overview of the sensitivity analysis for the elongation rate parameter. Box plots show the distribution of the slopes from linear regression (see **D**) for all fitted parameters and across all transcripts with multiple exons (n = 7935). Slopes were multiplied by 100 to represent relative change in percent. When the elongation rate is increased by 100%, the median change in the other parameters ranges from 4% for the membrane decay to 14% for the pre-mRNA processing rate. Transcripts that show nuclear degradation (n = 566) were excluded here due to simplicity reasons. Source data are available online for this figure.

 © The Author(s)

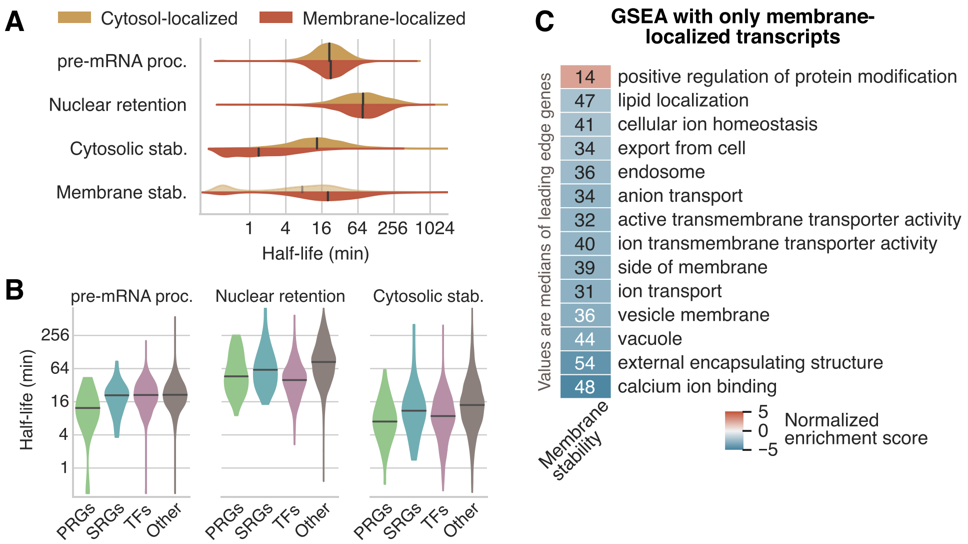

Figure EV4. Kinetic differences between different gene groups.

(**A**) Violin plots of pre-mRNA processing, nuclear retention, cytosolic and membrane half-lives for cytosol- (n = 7677) and membrane-localized (n = 1693) transcripts. (**B**) Violin plots of pre-mRNA processing, nuclear retention and cytosolic half-lives for primary response genes (PRGs, n = 56), secondary response genes (SRGs, n = 37), transcription factors (TFs, n = 709) and all other (n = 6875) transcripts. Only cytosol-localized transcripts were included. Definitions of PRGs and SRGs are taken from (Uhlitz et al, 2017). For definition of TFs, see Methods. (**C**) GSEA based on the GO using only membrane-localized transcripts and ranking by membrane decay. All significant terms (BH-adjusted *p* values <0.05) are shown. Color indicates normalized enrichment score. Annotated values are the median half-lives of leading edge genes of each term. Source data are available online for this figure.

© The Author(s)

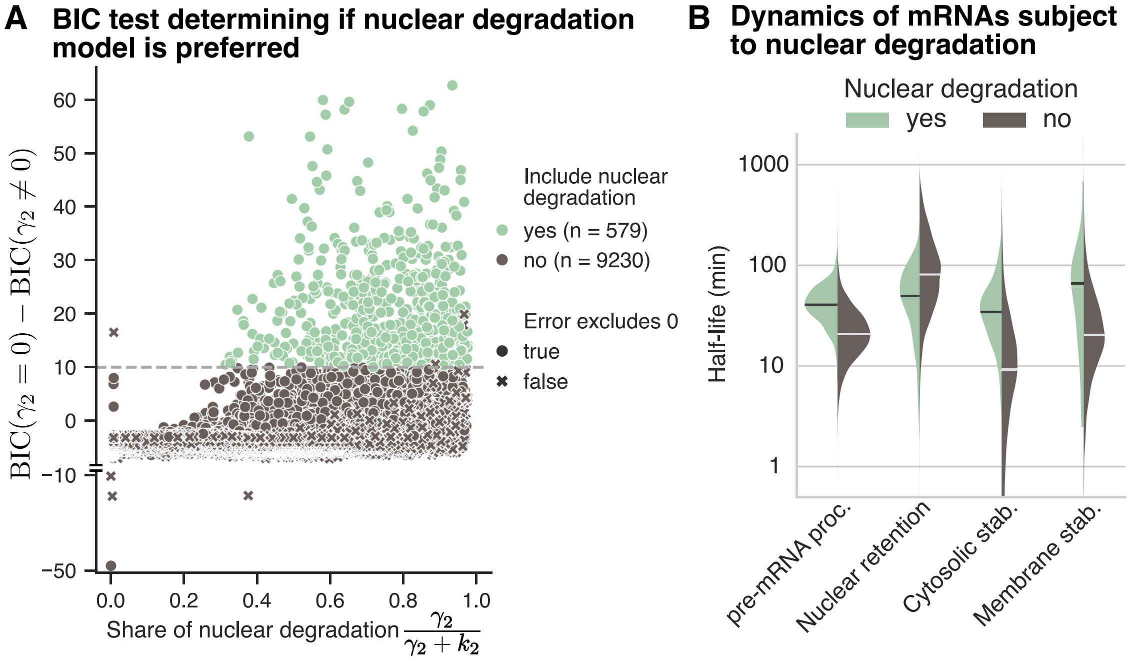

C GO analysis of nuclear decaying transcripts

Term name (intersection size)	BH-adjusted p-value
Base-exision repair (9)	0.05
DNA replication maintenance of fidelity (11)	0.05
Negative regulation of DNA metabolic process (16)	0.05
DNA damage response (60)	0.05
Toll like receptor signaling (7)	0.04

Figure EV5. Nuclear degradation of polyadenylated mRNAs.

(**A**) Comparison between models excluding and including the nuclear degradation parameter γ_2. Scatterplot shows the value difference between the Bayesian Information Criterion (BIC) from fit results of models excluding ($\gamma_2 = 0$) and including ($\gamma_2 \neq 0$) a nuclear decay parameter over the share of nuclear degradation at overall nuclear dynamics from the $\gamma_2 \neq 0$ model (n = 9809). For cytosol-localized transcripts, the BIC values from the 3-step models are compared. For undefined and membrane-localized transcripts, the BIC values from the 4-step models are compared. Based on (Raftery, 1995), the model including nuclear degradation is chosen if the BIC difference exceeds 10 (colored green), otherwise the model without nuclear degradation is chosen (colored black). Further selection is based on $\gamma_2 - s_{\gamma_2} > 0$ with s_{γ_2} being the standard error on the nuclear decay parameter. This test (true or false indicated by shape) checks that the 0 value is not included within the standard error and only if true the model with nuclear degradation is chosen. (**B**) Violin plots showing pre-mRNA processing, nuclear retention, cytosolic stability and membrane stability half-lives for transcripts modeled with (n = 579) and without (n = 9230) nuclear decay. Nuclear retention half-life is calculated as $\tau_2 = \frac{\ln 2}{k_2 + \gamma_2}$. Center lines of violin plots depict the median values. (**C**) Results from a GO analysis of all transcripts modeled with nuclear decay. All significant results (BH-corrected p value <0.05) are shown. Source data are available online for this figure.

 © The Author(s)
